# Supplementary material for: Regulatory Effects of Three-Dimensional Cultured Lipopolysaccharide-Pretreated Periodontal Ligament Stem Cell-Derived Secretome on Macrophages
Source: Int J Mol Sci. 2023 Apr 10;24(8):6981. doi: 10.3390/ijms24086981 (PMC10139044; doi:10.3390/ijms24086981)
Supplement: Supplementary file 1 [file ijms-24-06981-s001.zip › ijms-2252625-supplementary.pdf]

Supporting Information

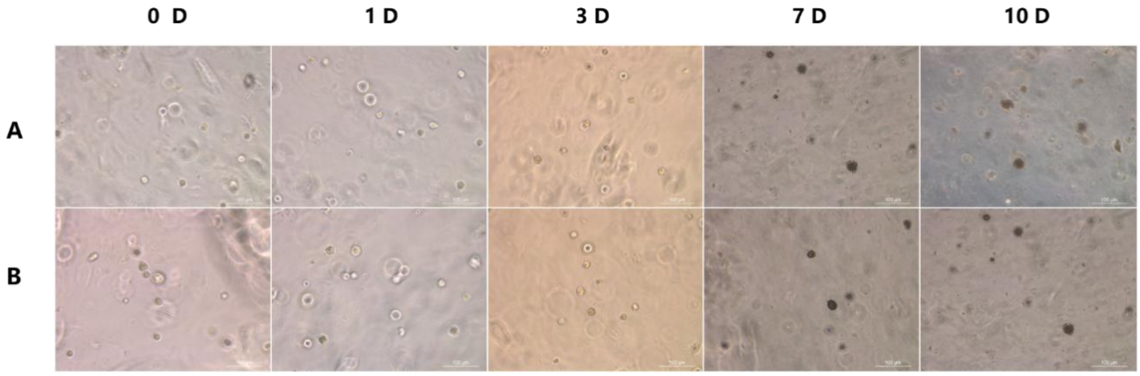

Figure S1. Growth states of PDLSCs and LPS pretreated PDLSCs in SupraGel under optical microscope. A, PDLSCs; B, LPS pretreated PDLSCs; D, Day; scale scale: 100 μm.

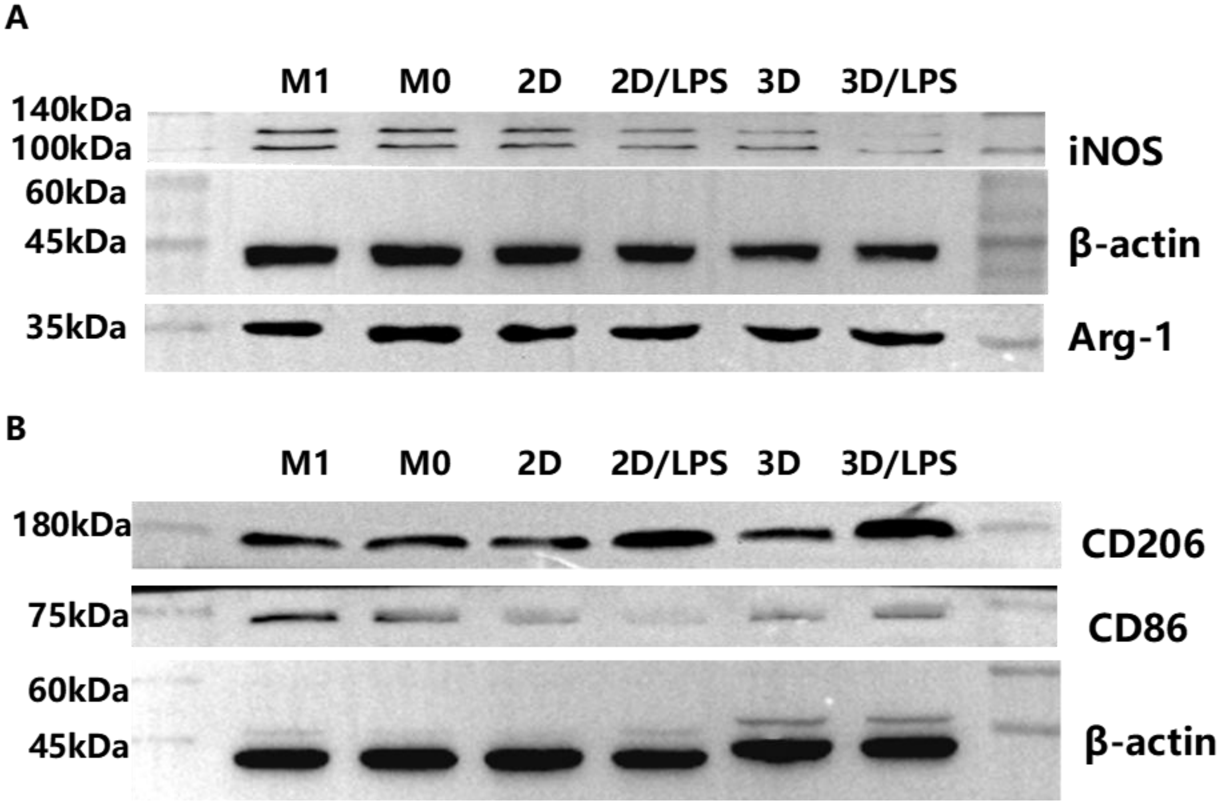

Figure S2. Original images of western blots

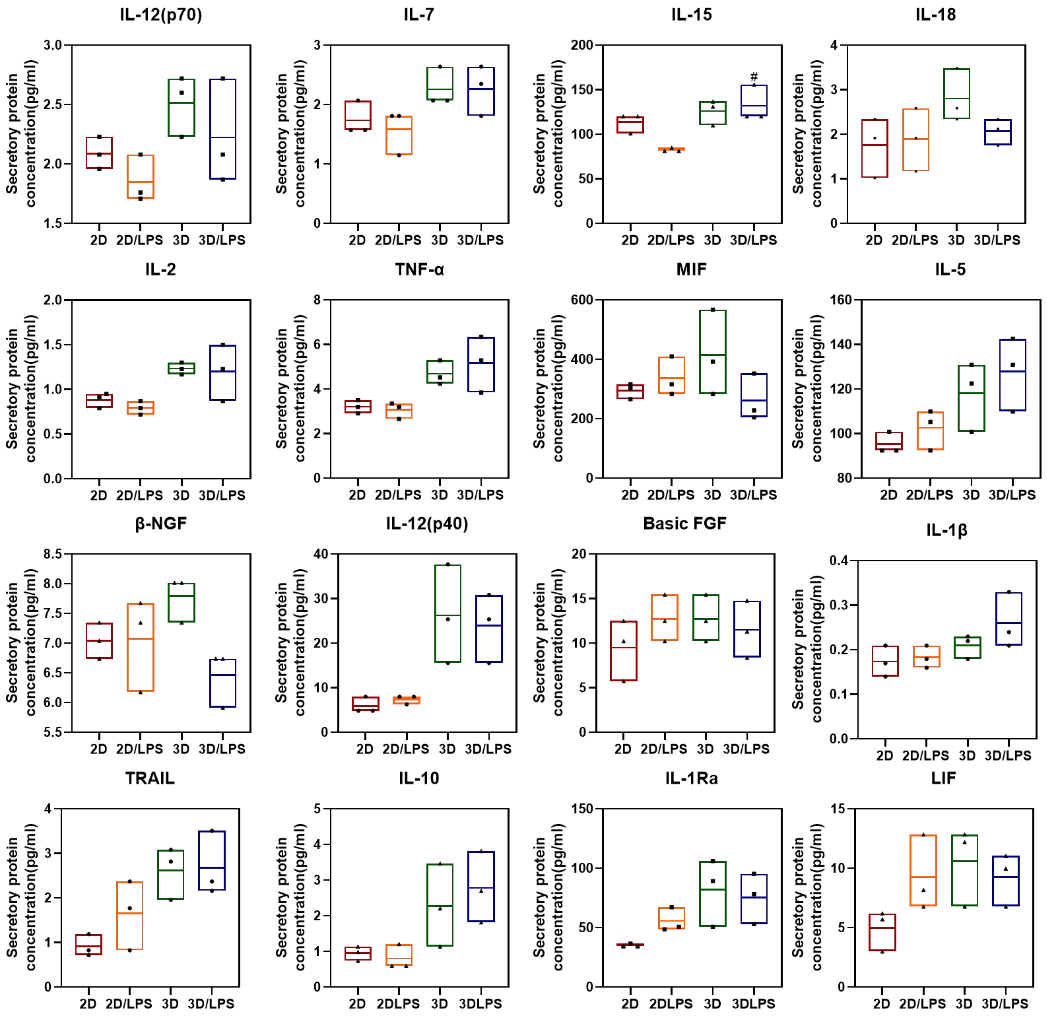

Figure S3. Cytokines with no significant changes of PDLSCs derived secretome after LPS and/or 3D culture treatment.
